# ESCRT-I Mediates FLS2 Endosomal Sorting and Plant Immunity

**DOI:** 10.1371/journal.pgen.1004035

**Published:** 2013-12-26

**Authors:** Thomas Spallek, Martina Beck, Sara Ben Khaled, Susanne Salomon, Gildas Bourdais, Swen Schellmann, Silke Robatzek

**Affiliations:** 1 The Sainsbury Laboratory, Norwich Research Park, Norwich, United Kingdom; 2 Department of Plant Microbe Interactions, Max Planck Institute for Plant Breeding Research, Cologne, Germany; 3 Botanical Institute, Biocenter Cologne, Cologne, Germany; Virginia Tech, United States of America

## Abstract

The plant immune receptor FLAGELLIN SENSING 2 (FLS2) is present at the plasma membrane and is internalized following activation of its ligand flagellin (flg22). We show that ENDOSOMAL SORTING COMPLEX REQUIRED FOR TRANSPORT (ESCRT)-I subunits play roles in FLS2 endocytosis in Arabidopsis. VPS37-1 co-localizes with FLS2 at endosomes and immunoprecipitates with the receptor upon flg22 elicitation. *Vps37-1* mutants are reduced in flg22-induced FLS2 endosomes but not in endosomes labeled by Rab5 GTPases suggesting a defect in FLS2 trafficking rather than formation of endosomes. FLS2 localizes to the lumen of multivesicular bodies, but this is altered in *vps37-1* mutants indicating compromised endosomal sorting of FLS2 by *ESCRT-I* loss-of-function. VPS37-1 and VPS28-2 are critical for immunity against bacterial infection through a role in stomatal closure. Our findings identify that VPS37-1, and likewise VPS28-2, regulate late FLS2 endosomal sorting and reveals that ESCRT-I is critical for flg22-activated stomatal defenses involved in plant immunity.

## Author Summary

Plants deploy plasma membrane immune receptors to survey their environment for potential threats. One of these receptors, FLAGELIN SENSING 2 (FLS2) recognizes bacterial flagellin (flg22) and thereby triggers a multitude of defense responses, enhancing immunity against infectious pathogens. Regulation of the subcellular localization of FLS2 is therefore an important aspect in plant disease resistance. FLS2 is known to shuttle between the plasma membrane and endosomal compartments but enters the late endosomal trafficking pathway upon ligand-dependent activation. A key question is the regulation of activated FLS2 in late endosomal trafficking. Here, we show that FLS2 is internalized into the lumen of multivesicular bodies and discovered by genetic inhibition that this step is regulated by components of the ENDOSOMAL SORTING COMPLEXES REQUIRED FOR TRANSPORT-I (ESCRT-I). Furthermore, we reveal that these ESCRT-I components play crucial roles in plant immunity impacting the flg22-triggered closure of stomata, prominent entry points of pathogenic bacteria, which occurred downstream of the known flg22 responses. These findings highlight the roles of endosomal trafficking in regulating FLS2 subcellular localization and plant immunity.

## Introduction

The metazoan and plant immune systems deploy pattern recognition receptors (PRRs) at the cell surface to sense a wide range of potentially pathogenic microbes through the presence of distinct pathogen-associated molecular patterns (PAMPs), conserved molecules displayed by microbes [1]. In plants, engagement of PRRs leads to the activation of signaling pathways that include mitogen-activated kinase (MAPK) cascades and a series of defense responses ranging from a rapid burst of reactive oxygen species (ROS) to deposition of callose [1]. FLAGELLIN SENSING 2 (FLS2) encodes the PRR that perceives the bacterial PAMP flagellin (flg22) and is required for immunity against bacteria [1]. Upon binding of flg22 to the receptor, FLS2 signaling pathways are activated by complex formation and phosphorylation between FLS2 and BRASSINOSTEROID INSENSITIVE 1 (BRI1)-ASSOCIATED KINASE 1 (BAK1) [2]. Activated FLS2 is internalized via the endocytic pathway raising the possibility that the pool of signaling FLS2 receptors at the plasma membrane is under tight regulation.

Following uptake from the plasma membrane, endocytosed FLS2 arrives at the SYP61-positive *trans*-Golgi network (TGN)/early endosomal (EE) compartment and the activated receptor is delivered to late endosomal compartments/multivesicular bodies (LE/MVB), from where it can be sorted for degradation [3], [4]. Endosomal sorting of vacuolar cargo involves the delivery of cargo to the LE/MVBs, and more precisely to the luminal vesicles of these compartments. This has been demonstrated in plants for only few plasma membrane proteins: PINFORMED 1 (PIN1), REQUIRES HIGH BORON 1 (BOR1), and BRI1 [5]–[10].

Ubiquitination of the cytosolic domains of plasma membrane proteins has emerged as a key signal for the delivery of these proteins to the LE/MVBs, and more precisely to the luminal vesicles of these compartments [5]–[8] Upon flg22 elicitation, two E3 ligases, PUB12/13, are recruited to FLS2 in a BAK1-dependent manner, and this promotes ubiquitination of FLS2 [11]. Posttranslational modification with ubiquitin targets proteins for MVB luminal sorting, which allows for the hypothesis that ubiquitination facilitates receptor internalization. This is supported by findings that loss-of-function mutations in *BAK1* and application of proteasome inhibitors block FLS2 endocytosis as well as several FLS2-mediated responses [2], [12].

The molecular machinery responsible for sorting ubiquitinated cargo to LE/MVBs is the ENDOSOMAL SORTING COMPLEX REQUIRED FOR TRANSPORT (ESCRT)-0, -I, -II, and –III [13]. The subunits of the ESCRTs are referred to as VACUOLAR PROTEIN SORTING (VPS), and with the exception of ESCRT-0, are highly conserved in plants [14]–[20]. The Arabidopsis VPS4 subunit homologue SKD1 (SUPPRESSOR OF K^+^ TRANSPORT GROWTH DEFECT 1) was reported to mediate vacuolar sorting of ubiquitinated cargo from the plasma membrane [21], [22], and the SKD1-interacting ESCRT-III related proteins CHARGED MULTIVESICULAR BODY PROTEIN (CHMP) 1A and B are involved in correct vacuolar sorting of PIN1, PIN2 and AUXIN RESISTANT 1 (AUX1) [9], [21]. However, surprisingly little is known about ESCRT-I-mediated cargo sorting and their function in plant processes.

Here, we found that endocytosed FLS2 co-localizes and co-purifies with the ESCRT-I subunit VPS37-1. In *vps37-1* knock-out plants, the endocytic pathway was normal but flg22-induced FLS2 endocytosis was reduced. We found that *vps37-1* mutants were affected not only in FLS2 internalization but also in the FLS2 localization to the lumen of MVBs indicating compromised vacuolar sorting of FLS2. Mutant *vps37-1* plants, and likewise *vps28-2*, supported enhanced growth of *Pseudomonas syringae* pv. *tomato* DC3000 (*Pto* DC3000), which was associated with compromised flg22-triggered stomatal closure. Neither VPS37-1 nor VPS28-2 is involved flg22-induced ROS burst, MPK activation or callose deposition linking the late endocytic trafficking of FLS2 specifically with defense-associated stomatal closure. Altogether, our findings provide novel aspects of FLS2 endocytosis and plant immunity mediated by ESCRT-I.

## Results

### Endocytosed FLS2 localizes to the inner lumen of MVBs

To dissect post-internalization trafficking of FLS2, we examined the localization pattern of FLS2-containing ARA6/RabF1-RFP and RFP-ARA7/RabF2b-labelled endosomes. Transient expression of RFP-ARA7/RabF2b by particle bombardment in Arabidopsis leaves and prolonged Wortmannin-treated ARA6/RabF1-RFP transgenic plants revealed different types of endosomal compartments labeled by these Rab5 GTPases; these treatments identified different populations of normal sized and enlarged, ring-like structures (Figure S1). Enlarged structures have been previously identified as a result of homotypic fusion of MVBs and this induced structure allows optical resolution of the outer membrane from the luminal cargo [23]–[26]. We measured RFP fluorescent intensity of transections across these endosomal compartments. These data showed that both Rab5 GTPases localize to the outer membrane of the ring-like structures (Figure S1; 1) indicating both ARA6/RabF1 and ARA7/RabF2b are located at the outer membranes of MVBs, consistent with previous reports [21], [24], [25].

**Figure 1:**
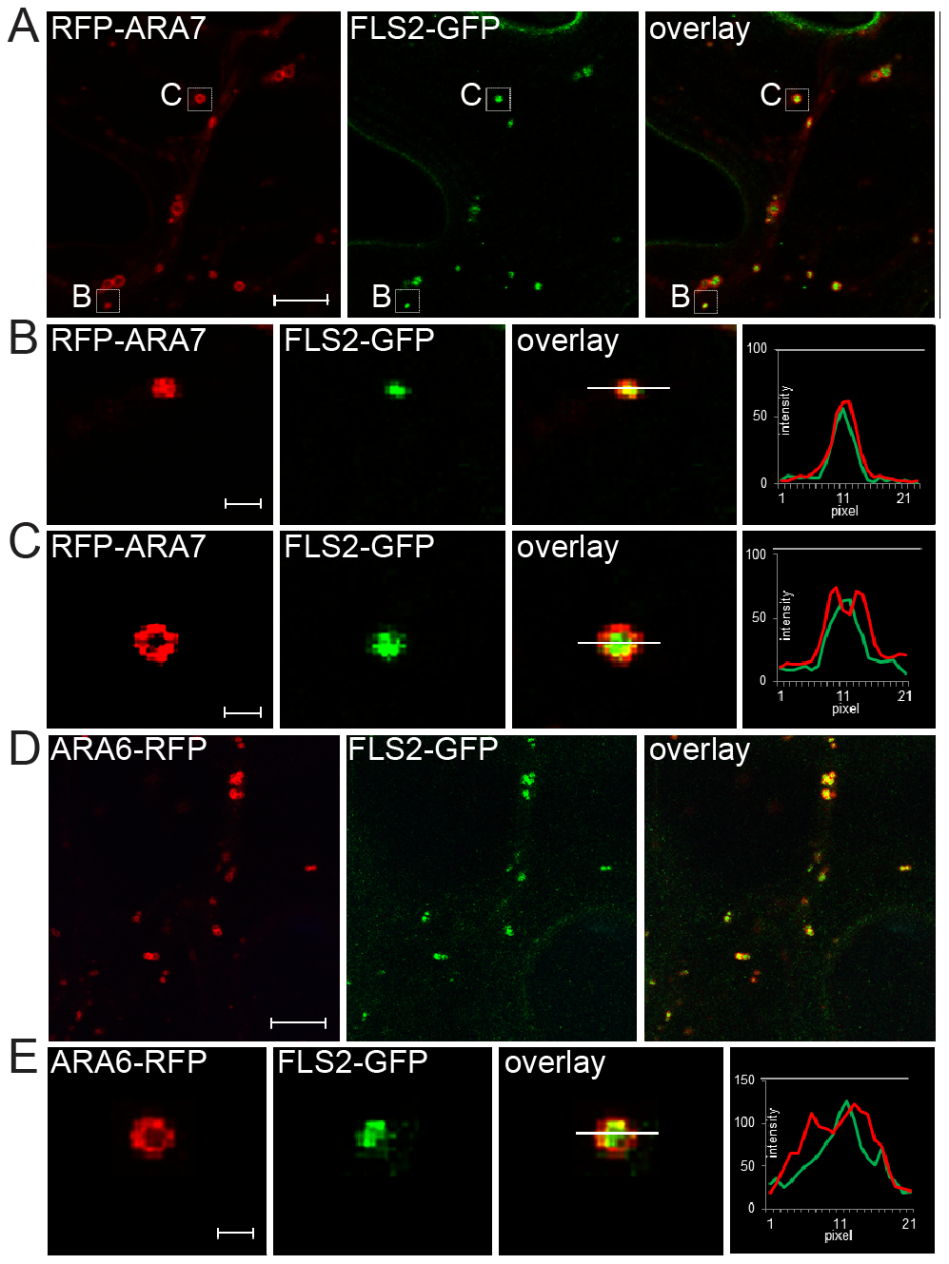
FLS2 is transported as luminal cargo of MVBs. (*A*) Standard confocal micrographs of FLS2-GFP transgenic leaf epidermal cells transiently expressing RFP-ARA7/RabF2b treated with 10 µM flg22 for 60 min; bar = 10 µm. (*B, C*) Detail images of co-localizing FLS2-GFP and RFP-ARA7/RabF2b as indicated by white boxes in *A*; bar = 1 µm. (*B*) Detail image of normal size MVB. (*C*) Detail image of the ring-like structure of enlarged MVBs. (*D*) Confocal micrographs of FLS2-GFP × ARA6/RabF1-RFP transgenic leaf epidermal cells treated with 10 µM flg22 for 45 min followed by 30 µM Wortmannin for 2 h; bar = 10 µm. (*E*) Detail images of co-localized FLS2-GFP and ARA6/RabF1-RFP at enlarged, ring-like structured MVBs; bar = 1 µm. Transections across endosomes used for fluorescence intensity measurements are indicated by white lines. The histograms show FLS2-GFP, RFP-ARA7/RabF1, and ARA6/RabF1-RFP fluorescent intensities depicted by green and red lines, respectively. Representative images of at least three experiments are shown.

To determine FLS2-GFP location at MVBs, we triggered FLS2 endocytosis and examined sub-compartment localization at enlarged MVBs. We first examined FLS2-GFP endosomes in transiently expressing RFP-ARA7/RabF2b leaf cells (Figure 1A). FLS2 and ARA7/RabF2b derived GFP and RFP signals were overlapping and showed similar single peak GFP/RFP fluorescent intensity curves when measured from normal sized endosomal compartments (Figure 1B). At enlarged MVBs, endosomal FLS2 and ARA7/RabF2b showed different localizations. The FLS2-GFP signal was primarily present in the lumen as a filled circle within the ring-like structure marked by RFP-ARA7/RabF2b signal (Figure 1C). This is also illustrated by the GFP/RFP fluorescent intensity curves of transections across these endosomal compartments revealing GFP peaking between two RFP peaks. When we performed prolonged Wortmannin treatment of FLS2-GFP×ARA6/RabF1-RFP plants, the FLS2-GFP signal was similarly concentrated within the ARA6/RabF1-RFP signal in the lumen of these ring-like structures (Figure 1D, 1E). Thus, as with BRI1, BOR1 and other plasma membrane vacuolar cargo, FLS2 endocytosis via the late endosomal pathway involves trafficking to the lumen of MVBs [9], [10], [24].

### FLS2 associates and co-localizes with activated VPS37-1 at endosomes

**Figure 2:**
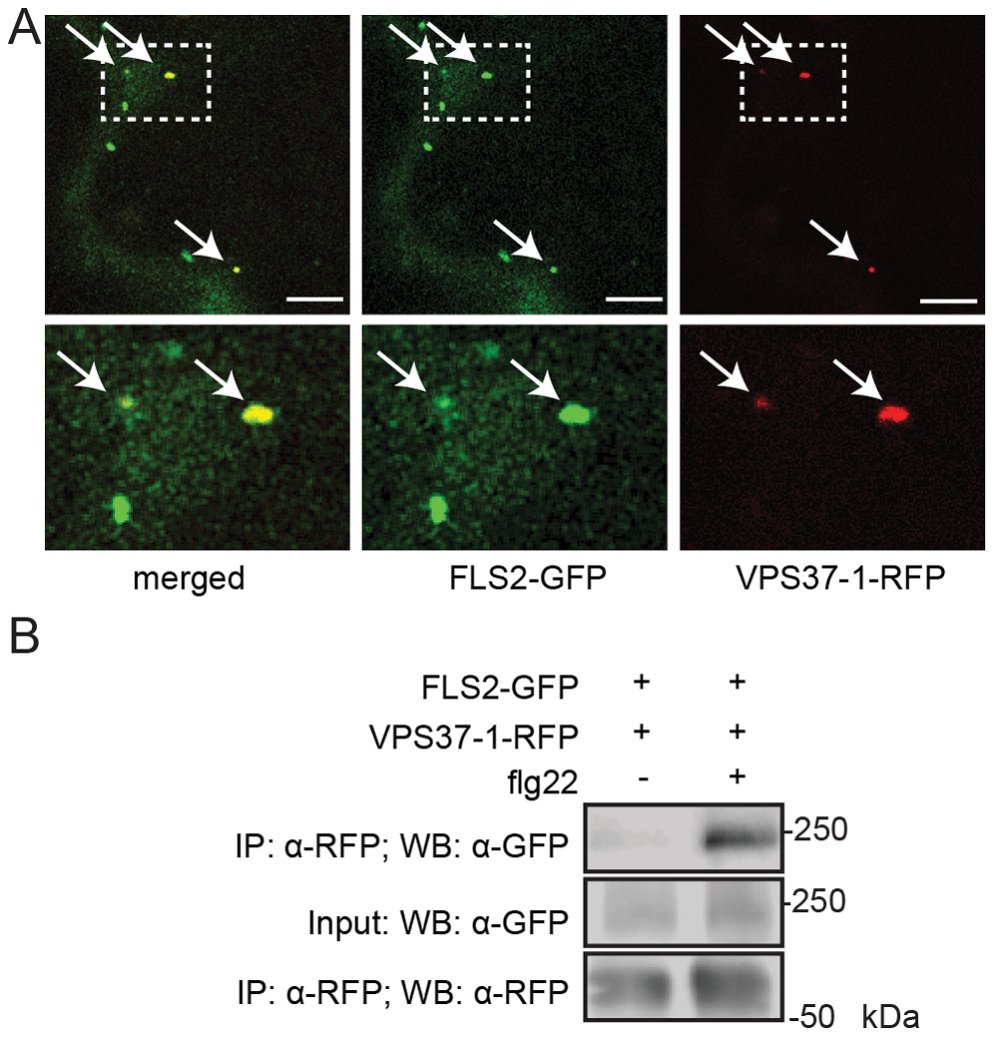
FLS2 is present at ESCRT-I-positive compartments and forms an inducible complex with VPS37-1. (*A*) Standard confocal micrographs of show optical sections of *N. benthamiana* leaf epidermal cells transiently expressing FLS2-GFP and RFP-VPS37-1 treated with 100 µM flg22 for 100 min. Upper panel represents overview image, lower panel shows detail image of co-localization between FLS2-GFP and RFP-VPS37-1 at vesicles (indicated by arrows); bar = 10 µm. (*B*) Co-expressed FLS2-GFP and RFP-VPS37-1 were subjected for immunoprecipitation analysis. RFP-VPS37-1 was able to co-purify FLS2-GFP upon flg22 stimulus. These experiments have been repeated three times.

FLS2 is ubiquitinated in response to flg22 elicitation and sorting of ubiquitinated plasma membrane proteins into luminal vesicles of MVBs is a process mediated by the ESCRT machinery. Therefore, we sought to determine whether ESCRT components play a role in FLS2 endocytosis and trafficking. ESCRT-I is a heterotrimeric complex composed of the subunits VPS23/ELC, VPS28 and VPS37 [19]. In Arabidopsis, the VPS28-1 subunit was recently shown to localize to the TGN/EE, from which MVB maturation could be observed [8]. As mutants in *VPS23/ELC* were in Ws-0 background, which lacks a functional *FLS2* gene [19], [27], we focused on VPS37-1 and VPS28-2 [18]. To determine if VPS37-1 and VPS28-2 are involved in FLS2 endosomal trafficking, we performed *in planta* co-localization experiments following flg22 elicitation. Endosomal FLS2-GFP partially co-localized with RFP-VPS37-1 and RFP-VPS28-2, respectively (Figure 2A, S2A), a similar pattern compared to the partial co-localization of FLS2-GFP with ARA7/Rab2Fb- and ARA6/RabF1-positive endosomes at early time points after flg22 treatment in Arabidopsis [3]. This suggests that FLS2 traffics via ESCRT-I-positive compartments along its endocytic route.

To investigate whether co-localization is indicative of an interaction between ESCRT-I components and FLS2, we performed co-immuniprecipitation analysis with VPS37-1. In the absence of flg22, FLS2-GFP was detected only in minor amounts of immunoprecipitated RFP-VPS37-1 (Figure 2B). By contrast, the levels of FLS2-GFP were significantly increased in immunoprecipitated RFP-VPS37-1 upon flg22 elicitation. These results indicate that FLS2 forms an inducible complex with the ESCRT-I subunit VPS37-1 coinciding with their shared endosomal localization.

### FLS2 endocytosis but not formation of endosomes depends on *VPS37-1*

**Figure 3:**
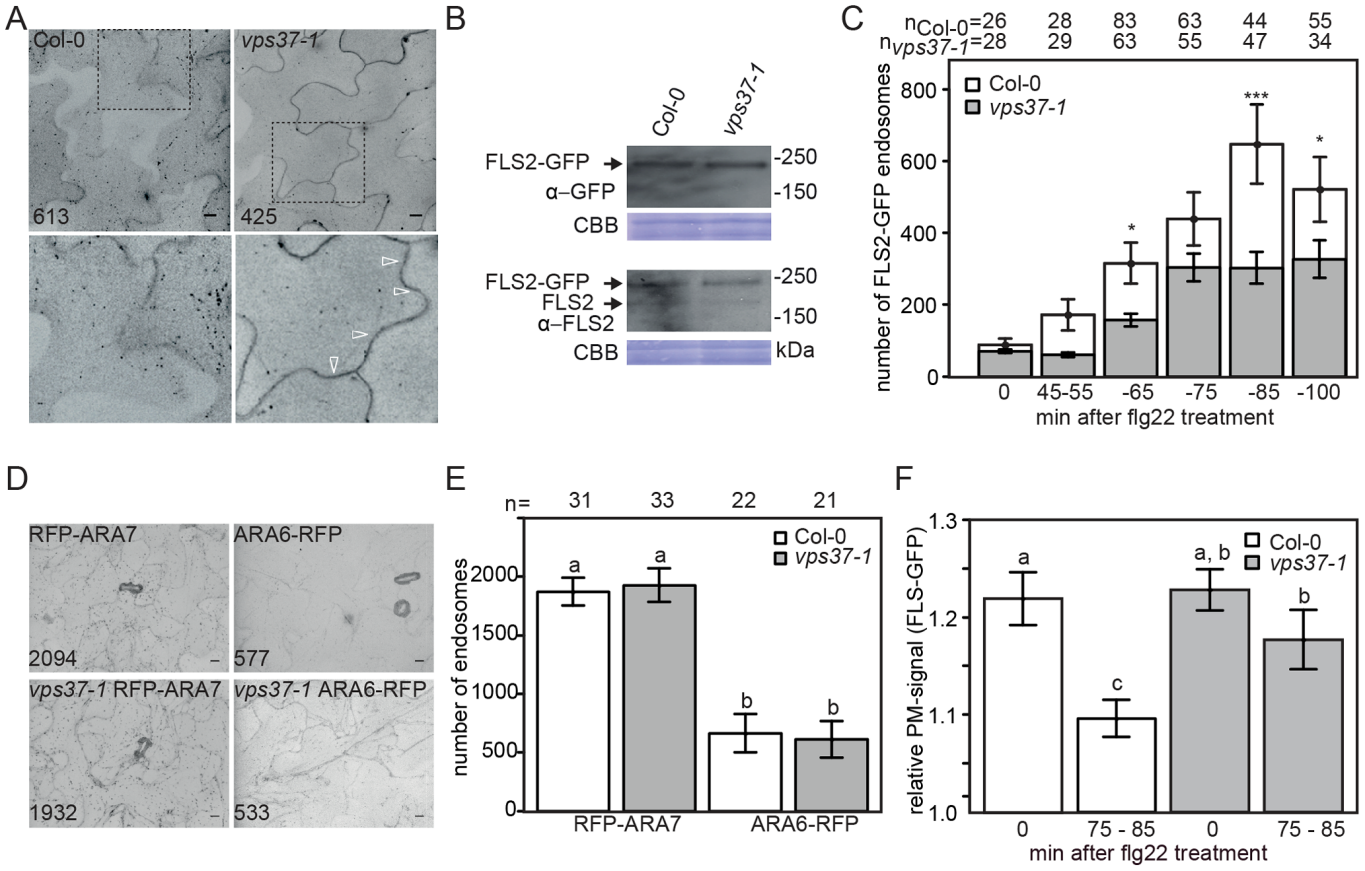
Flg22-induced endocytosis of FLS2 is compromised in *vps37-1* mutants but not steady-state ARA6/RabF1 and ARA7/RabF2b endosomal numbers. (*A*) High-throughput confocal micrographs show Arabidopsis cotyledon cells of the indicated FLS2-GFP transgenic lines treated with 10 µM flg22 for 80 min. Detail pictures show FLS2-GFP endosomes (indicated with arrows); bar = 10 µm; numbers indicate total endosomes. (*B*) Immunoblot detection of endogenous FLS2 and transgenic FLS2-GFP protein accumulation in homozygous Col-0 and *vps37-1* plants. Coomassie brilliant blue (CBB) is used as loading control. (*C*) Quantification of FLS2-GFP endosomal numbers upon treatment with 10 µM flg22 at the indicated times and genotypes. Error bars represent mean values +/− SE; n>26<83 images. Asterisks indicate statistical significance of p<0.05 (*) and p<0.001 (***). (*D*) High-throughput confocal micrographs show Arabidopsis cotyledon cells of the indicated ARA6/RabF1-RFP and RFP-ARA7/RabF2b transgenic lines; bar = 10 µm; numbers indicate total endosomes. (*E*) Quantification of ARA6/RabF1-RFP and RFP-ARA7/RabF2b endosomal numbers. Error bars represent mean values +/− SE; n images. Letters indicate statistical significance of p<0.05. (*F*) Quantification of the FLS2-GFP fluorescence signal at the plasma membrane upon treatment with 10 µM flg22 at the indicated times and genotypes. Error bars represent mean values +/− SE; n = 50. Letters indicate statistical significance (p<0.05, ANOVA and Tukey's honestly test). These experiments have been repeated three times.

The observation that activated FLS2 co-localizes and forms a complex with VPS37-1 suggest that this ESCRT component plays a critical role in FLS2 endocytosis and trafficking. To investigate this role, we crossed FLS2-GFP into *vps37-1* mutants and examined flg22-induced FLS2 endocytosis. Steady-state expression of FLS2-GFP and localization at the plasma membrane was wild type-like (Figure 3A, 3B, S3A), but following flg22 treatment we observed lower numbers of FLS2-GFP endosomes when compared to wild type plants (Figure 3A). Quantification of flg22-induced endosomes by high-throughput imaging demonstrated that despite a general increase in endosome numbers over time, the total numbers of FLS2-GFP endosomes in the 55–100 minutes following flg22 elicitation was significantly lower in the mutants compared to wild type plants (Figure 3C). FLS2-GFP endosomes present in *vps37-1* mutants showed co-localization with RFP-ARA7/RabF2b indicating that, while fewer in number, these vesicles are endosomal compartments of the late FLS2 endocytic trafficking route (Figure S3C; [3]).

Reduced flg22-induced internalization of FLS2 was also observed in *vps28-2* mutants (Figure S2E). However, we cannot rule out the possibility that this is caused by reduced FLS2-GFP protein levels in this background, because in several attempts of crosses and transformation we were unable to obtain *vps28-2* lines expressing FLS2-GFP at similar levels than wild type, though steady-state expression of endogenous FLS2 was unaltered (Figure S2F). Nevertheless, our experiments collectively show that FLS2-positive endosomes are decreased in ESCRT-I mutant plants implying a defect in trafficking.

To ensure that the reduced number of FLS2 endosomes observed in *vps37-1* mutants is not a result of global changes to the endosomal populations, we compared ARA7/RabF2b- and ARA6/RabF1-positive endosomes in mutant lines and wild type. We crossed RFP-ARA7/RabF2b and ARA6/RabF1-RFP into the *vps37-1* background and endosome quantification by high throughput imaging indicated that there was no difference between the *vps37-1* mutant and wild type (Figure 3D, 3E). This data suggest that steady-state endosomal numbers, at least as revealed by these two EE/LE/MVB markers, are not affected in *vps37-1* plants and that the reduction in FLS2 endosomes likely reflects a defect in specific endosomal trafficking of the receptor.

The observed effect on FLS2 endocytosis in *vps37-1* mutants could result from inhibited FLS2 trafficking at the plasma membrane. To test this hypothesis, we measured the fluorescence intensities of plasma membrane FLS2-GFP. In contrast to wild type plants, the fluorescence intensity of plasma membrane-resident FLS2-GFP in *vps37-1* mutants decreased to a much lesser extent after flg22 treatment (Figure 3F). Depending on its activation status, FLS2 is internalized from the plasma membrane into two distinct trafficking routes [3]. We therefore examined whether *vps37-1* mutants were affected in endosomal recycling of the non-activated FLS2 receptor. Treatment with Brefeldin A (BFA) caused the accumulation of FLS2-GFP in so-called BFA-bodies, stained by the endocytic tracer FM4-64 (Figure S3D). When *vps37-1* leaves were treated with both BFA and flg22, FLS2-GFP-positive endosomes were detected around the BFA-body (Figure S3D), as previously described in wild type [3]. These observations show that endosomal recycling of the non-activated receptor is not regulated by VPS37-1 in agreement with a role of ESCRT in the delivery of cargo for vacuolar degradation. Additionally, these observations might indicate that flg22-induced FLS2-positive endosomes maintain trafficking along the late endosomal pathway rather than entering recycling trafficking.

### Impact of *vps37-1* on FLS2 endocytosis is related to MVB sorting

**Figure 4:**
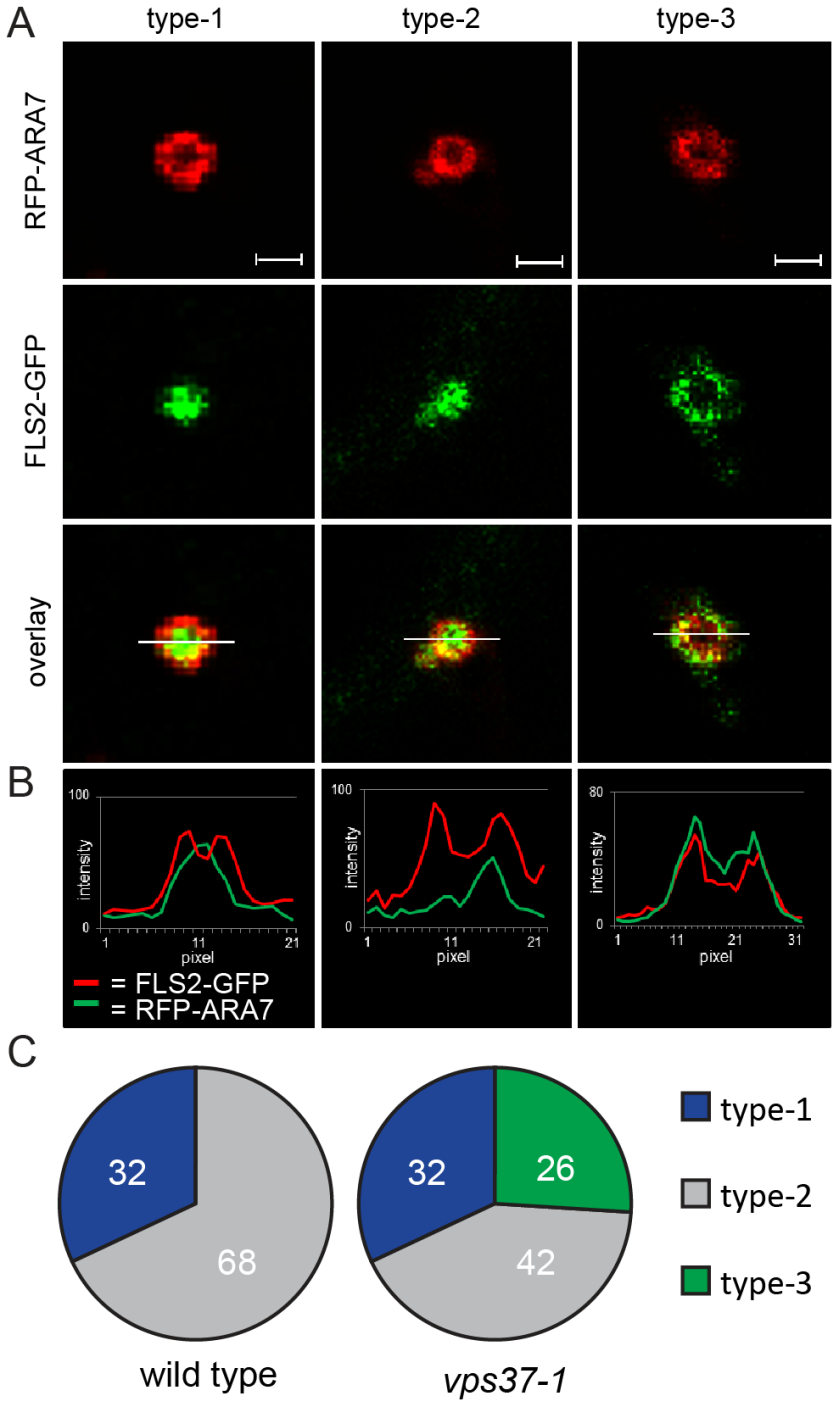
VPS37-1 contributes to FLS2 sorting in MVBs. (*A*) Detail confocal images show three types of FLS2-GFP and RFP-ARA7/RabF2b co-localization at enlarged, ring-like structure MVBs in FLS2-GFP transgenic wild type and *vps37-1* leaf epidermal cells transiently expressing RFP-ARA7/RabF2b treated with 10 µM flg22 for 60 min; bar = 1 µm. Representative images of four independent experiments are shown. Transections across endosomes used for fluorescence intensity measurements are indicated by white lines. (*B*) The histograms show FLS2-GFP and RFP-ARA7/RabF1 fluorescent intensities depicted by green and red lines, respectively. (*C*) Quantification of the three types of FLS2-GFP and RFP-ARA7/RabF2b co-localization at enlarged, ring-like structure MVBs in the indicated genotypes. In total, n = 50 (wild type) and n = 65 (*vps37-1*) enlarged, ring-like structure MVBs were analysed by fluorescence intensity measurements.

Our observation revealed that flg22-induced endocytosed FLS2 localizes to the lumen of MVBs. Since the primary role of the ESCRT machinery is associated with sorting vacuolar cargo at MVB compartments [13], [15], we tested whether FLS2 localization at MVBs is altered in *vps37-1* mutants. Using particle bombardment, we transiently expressed RFP-ARA7/RabF2b in leaves of FLS2-GFP × *vps37-1* plants and examined the RFP and GFP fluorescence signals at enlarged MVBs. We observed three different types of fluorescent patterns (Figure 4A). At type-1 enlarged MVBs, the FLS2-GFP signal was primarily present in the lumen as a filled circle within the RFP-ARA7/RabF2b-labeled ring-like structure. The FLS2-GFP signal at type-2 enlarged MVBs was present in the lumen but also partially co-localized with the RFP-ARA7/RabF2b signal at the outer membrane of the ring-like structure. By contrast, the FLS2-GFP signal primarily co-localized with the RFP-ARA7/RabF2b signal at the outer membrane of type-3 enlarged MVBs and was almost not present in the lumen of its ring-like structure. This is further evident from measurements of the GFP/RFP fluorescent intensity curves of transections across these three types of enlarged MVBs (Figure 4B). These three types of FLS2 localization at enlarged MVBs might represent different stages of sorting activated FLS2 from the outer MVB membrane to the inner lumen.

We quantified the occurrence of type-1, type-2 and type-3 enlarged MVBs in wild type and *vps37-1* plants. Strikingly, type-3 enlarged MVBs were only detected in *vps37-1* mutants (Figure 4C). We counted about 26% (n = 65) type-3 enlarged MVBs in this background, whereas we could not identify this type of enlarged MVB in the wild type (Figure 4C). Type-2 enlarged MVBs were observed in both wild type and *vps37-1* plants at about 32%. Type-1 enlarged MVBs were also present in both genotypes, but a different numbers. Wild type plants showed about 68% type-1 enlarged MVBs, which was reduced to about 42% in *vps37-1* mutants. This result shows that FLS2 localization at MVBs is altered in *vps37-1* mutants and indicates that VPS37-1 impacts FLS2 endosomal trafficking associated with sorting processes from the outer membrane to the lumen of MVBs. However, overall endocytic trafficking from the plasma membrane to the vacuole was not impaired in *vps37-1* plants, because we did not observe any significant difference in the timing of FM4-64 uptake and vacuolar staining in both genotypes (Figure S3E). Together with no obvious developmental phenotype of *vps37-1* plants, this indicates that endocytic trafficking might affect a specific subset of vacuolar cargo in these ESCRT-I mutants.

### *VPS37-1* is required for immunity against bacterial infection

**Figure 5:**
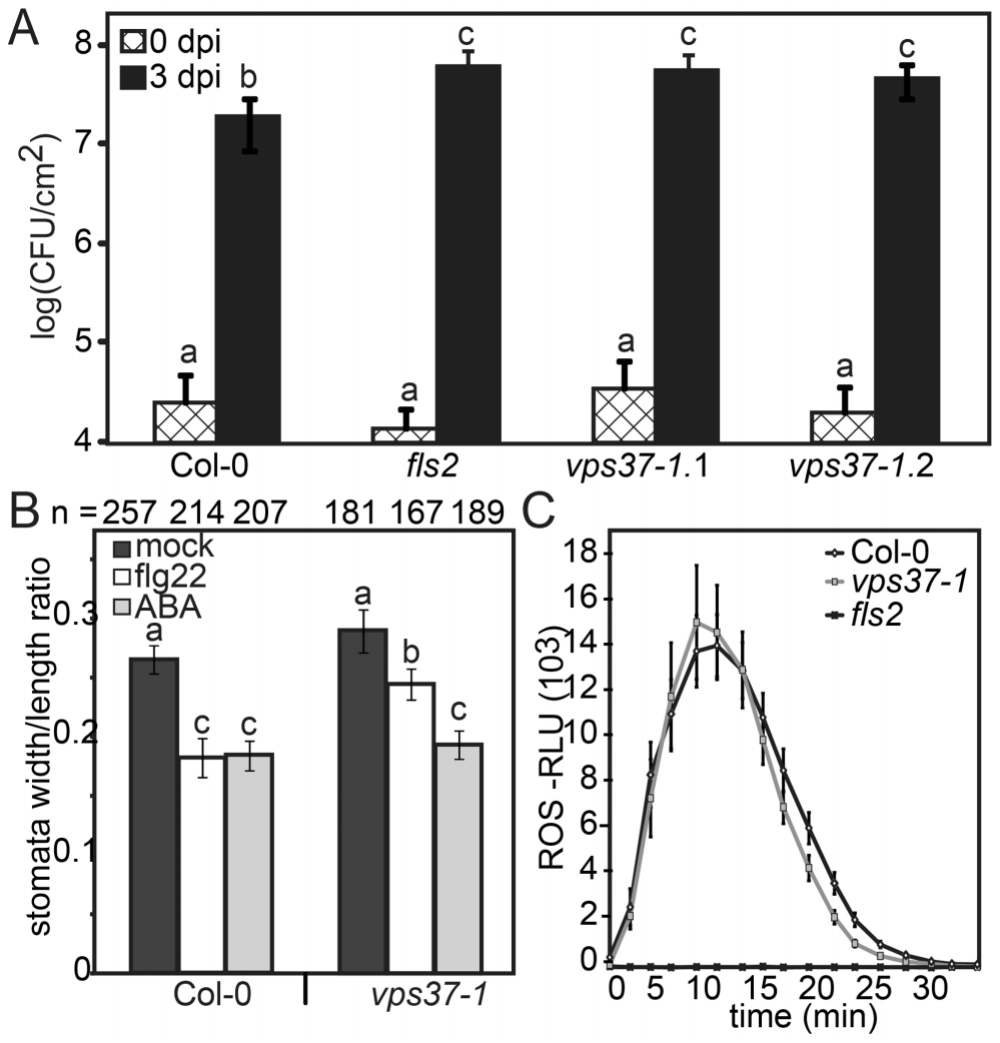
Knock-out *vps37-1* mutants are impaired in immunity. (*A*) Four weeks-old plants of the indicated genotypes were surface inoculated with *Pto* DC3000 and bacterial multiplication was monitored at 0 (4 hrs) and 3 dpi. Shown are mean values +/− SE; n = 6; asterisks indicate significant differences p<0.01 based on ANOVA and Tukey's honestly test. (*B*) Stomatal apertures were measured following treatments with water (mock), 10 µM flg22, 5 µM ABA for 90 min. Bars represent mean values +/− SE; n>138<268 stomata. All statistical analysis is based on ANOVA and Tukey's honestly test and letters indicate statistical significance of p<0.05. (*C*) ROS generation in leaf discs of the indicated genotypes triggered by 10 µM flg22 over time. Error bars represent mean values +/− SE; n = 16. All experiments have been repeated at least three times.

Given that FLS2 endocytosis is dependent on ESCRT-I this provides an opportunity to dissect which flg22-triggered defense responses are associated with changes in FLS2 trafficking. To test this hypothesis, we examined growth of *Pto* DC3000 in two independent alleles of *vps37-1* and *vps28-2* T-DNA insertion lines. Following spray infection of virulent *Pto* DC3000, bacterial growth was significantly higher in these ESCRT-I mutants compared to Col-0 wild type (Figure 5A, S2B) confirming a role for ESCRT-I in bacterial defense.

A critical layer of immunity control against bacterial infection involves the closure of stomata triggered by PAMPs, an important component of FLS2-mediated immunity that restricts pathogen entry at the pre-invasive level [28]. To address stomatal responses in *vps37-1* and *vps28-2*, we treated cotyledons with flg22 and measured the stomatal apertures. This was compared with aperture measurements under control conditions and upon incubation with abscisic acid (ABA), a well-described hormonal trigger of stomatal closure in drought stress [29], [30]. We observed that flg22-induced stomatal closure was significantly impaired in *vps37-1* and *vps28-2* plants (Figure 5B, S2C). Because ABA-triggered stomatal closure was wild type-like in all tested genotypes, these results indicate that flg22-triggered stomatal responses were specifically affected (Figure 5B, S2C).

The flg22-induced oxidative burst, an early PAMP response mediated by the NADPH oxidase RbohD, is required for stomatal closure [1], [31] and therefore we examined this response in the *ESCRT-I* mutants. Wild type, *vps37-1* and *vps28-2* plants displayed comparable ROS production upon flg22 elicitation (Figure 5C, S2D). Thus, in *vps37-1* and *vps28-2* plants, the flg22-induced stomatal closure is likely affected at a step downstream of the oxidative burst.

FLS2 signaling activates MPK3 and MPK6, both of which were recently reported to control stomatal closure triggered by flg22 [32]. Flg22-activation of MAP kinases was unaltered in *vps37-1* mutants when analyzed in whole plant extracts (Figure S3A). We additionally tested flg22-elicited callose deposition, a late PAMP response, because callose plays roles in plant immunity and has also been implicated in the mechanism of stomatal closure [33]. No significant difference in callose deposition was observed between flg22-treated wild type and *vps37-1* leaves (Figure S3B). Taken together, we conclude that VPS37-1 and VPS28-2 function is required for full immunity against bacterial infection while not broadly affecting known FLS2-mediated responses. These ESCRT-I subunits are required for flg22-triggered stomatal closure through a mechanism independent of known components of the FLS2 pathway.

## Discussion

Components of the ESCRT machinery are known to localize at the outer membrane of MVBs coupling the formation of MVB luminal vesicles and sorting cargoes for vacuolar degradation [13], [21], [34]. Despite their essential role in delivering endocytosed cargo to the vacuole, it is surprising that knowledge about the molecular interaction between ESCRT components and cargoes is limited. In plants, so far only the ESCRT-III-related CHMP1A and CHMP1B have been linked with sorting the *in planta* cargoes PIN1, PIN2, and AUX1 [9]. All of these cargoes are internalized by the recycling endosomal pathway and are mis-localized in *chmp1a × chmp1b* mutants. Here, we established that VPS37-1 and VPS28-2, two ESCRT-I components, play a role in flg22-induced late endosomal trafficking of FLS2. Our data suggest this is critical for sorting the receptor from the outer membrane to the lumen of MVBs but does not impact recycling endocytosis, ARA6/RabF1- and ARA7/RabF2b-positive endosome numbers nor FM4-64 trafficking to the vacuole.

TOLL and the TOLL-LIKE RECEPTORs (TLRs) are essential PRRs of the metazoen immune systems, and like FLS2, TOLL and TLRs localize to endosomes [35], [36]. Ligand-activated TLR4 is endocytosed for degradation and associates with the ESCRT-0 subunit HRS at MVBs [37]. Likewise, endosomal TOLL is found in a complex with HRS, and knocking down HRS inhibited the degradation of CACTUS downstream of TOLL suggesting that endocytosis contributes to proper TOLL signaling [35]. Our experiments indicate that MVB sorting is disrupted by the *vps37-1* knock-out mutation. Indeed, in the absence of VPS37-1, FLS2-GFP was observed at the MVB outer membrane. However, despite this altered sorting of FLS2-GFP, we did not detect any FLS2-GFP at the tonoplast as reported for the PIN proteins in *chmp1a × chmp1b* mutants [9]. This could suggest that activated FLS2 is inefficiently sorted to the MVB lumen and this impaired or delayed process allowed the visualization of these type-3 endosomes. Inefficient and/or delayed FLS2 endosomal sorting is in agreement with the greater abundance of FLS2-GFP at the plasma membrane observed in *vps37-1* mutants, likely resulting from reduced internalization, compared to wild type.

Mutations in ESCRT complex subunits and other related proteins often induce severe developmental defects [9], [19], [38]. However, neither *vps28-2* nor *vps37-1* showed any obvious developmental phenotype indicating the possibility of genetic redundancy with their respective closely homologous genes *VPS28-1* and *VPS37-2*. Significantly, both mutants were compromised in immunity against a bacterial pathogen. This and our observations that VPS37-1 and FLS2 are found in the same protein complex after flg22 elicitation imply that VPS37-1, and likewise VPS28-2, are involved in a mechanism to control the vacuolar sorting of activated FLS2. It is however possible that ESCRT-I components regulate this process for a number of plasma membrane proteins through a similar mechanism.

Recent reports describe both ubiquitin- and ubiquitin-independent degradation of vacuolar cargo in plants [8], [24]. Ubiquitination of endocytosed cargo has been identified for plasma membrane proteins including FLS2, BOR1 and PIN2 [6], [39]. Both the proteasome inhibitor MG132 and a mutation of a putative PEST degradation signal motif in the FLS2 kinase domain inhibit flg22-induced internalization of FLS2 providing indirect evidence that ubiquitination acts as a signal of FLS2 endocytosis [12], [40]. Further studies will reveal whether ESCRT-mediated sorting of endosomal FLS2 depends on ubiquitination, and whether this requires the function of the PUB12/13 E3 ligases that ubiquitinate activated FLS2 via interacting with BAK1 [11]. However, *pub12/13* mutants show increased flg22 responses and resistance to *Pto* DC3000 infection, in contrast to our findings of enhanced susceptibility in the *vps28-2* and *vps37-1* mutants. Therefore it is possible that regulation of FLS2 by PUB12/13 is involved at a different step of the endocytosis pathway.

Another purpose of endocytic trafficking of plasma membrane proteins is to control the activated signaling pathways. To date, the intersection between receptor-mediated endocytosis and signaling in plants has been best studied for BRI1 [10], [41], [42]. A recent report blocking BRI1 internalization by targeting clathrin demonstrates that BRI1 mostly signals from the plasma membrane contrasting an earlier study revealing BRI1 signaling from endosomes [41], [42]. There is accumulating evidence that effects on the downstream responses can differ depending at which level the inhibition of endocytic trafficking occurs. For example, blocking the internalization of the EPIDERMAL GROWTH FACTOR RECEPTOR (EGFR) at the plasma membrane resulted in an increase of the transcriptional response [43], [44], whereas inhibition of EGFR endosomal sorting by VPS4 knock-down did not increase or alter the overall pattern of the EGF transcriptional response but rather specifically affected a subset of signaling pathways [43], [45]. Relatedly, we found that interference with FLS2 sorting at the level of MVBs compromised specifically FLS2-mediated stomatal closure (Figure S4), which is consistent with the notion that FLS2 activates separate signaling branches [46], [47].

In this study we identified that VPS37-1 and VPS28-2 are required for flg22-induced stomatal closure but not for a range of other flg22-induced defense responses. This implicates post-internalization sorting of FLS2 specifically in PAMP-triggered stomatal closure and thus identifies a role for FLS2 endocytosis in bacterial immunity. Interestingly, immunity in *vps37-1 and vps28-2* was not compromised when bacteria were inoculated into the leaf tissue providing further evidence for a prominent role of these ESCRT-I components in stomatal immunity (data not shown). ESCRT-I-mediated stomatal closure is not sensitive to ABA treatment confirming it is part of a FLS2-specific mechanism that involves the late endosomal pathway. Although there is substantial knowledge about ABA-mediated control of stomatal apertures, the exact pathways underlying PAMP-triggered stomatal closure have only been partially described [48]. Current knowledge suggests branching and conversion of separate pathways and some role for ABA in the regulation of stomatal immunity [28], [32]. Flg22-induced stomatal closure in the ESCRT-I mutants is affected downstream of RbohD, MPK3 and MPK6, suggesting regulation could occur at the level of ion channel activity. The K^+^ channel KAT1 is required for ABA-mediated stomatal closure and also undergoes recycling endocytosis in response to ABA [49]. However, ABA-triggered stomatal closure and endosomal recycling was not affected in the ESCRT-I mutants, thereby indicating that VPS37-1 and VPS28-2 might function in a yet unknown manner in biotic stomatal aperture control. This line with the notion that ABA is primarily sensed by cytosolic receptors while FLS2 is membrane-bound (27,29), and it will be interesting to unravel the molecular mechanism of how endosomal trafficking intersects with stomatal immunity in the future.

This study has revealed that ESCRT-I plays a critical role in late endocytic sorting of FLS2 at the MVB. ESCRT-I is essential for plant immunity to a bacterial pathogen, specifically via flg22-induced stomatal closure. This identifies a role for FLS2 late endocytic trafficking in the intitiation of specific defense responses and the existence of an independent mechanism for flg22-induced stomatal closure.

## Materials and Methods

### Plant materials and growth conditions

*Arabidopsis thaliana* plants were grown on general soil (Arabidopsis mix, John Innes Centre, Norwich), for infection assays on Jiffy pellets (Jiffy Products, Norway), or for sterile conditions on Murashige and Skoog medium under 10 hours or 16 hours of light at 20–22°C and 65% humidity. Col-0/FLS2-GFP, ARA6/RabF1-RFP, and RFP-ARA7/RabF2b lines and *fls2*, mutants have been described previously [3], [12], [19], [21], [27], [50]. Homozygous T-DNA insertion *vps28-2*, *vps37-1* (*vps37-1.1*) and *vps37-1.2* lines were obtained from SAIL, SALK and GABI-KAT populations (Figure S4). Homozygous FLS2-GFP, ARA6/RabF1-RFP, RFP-ARA7/RabF2b lines in *vps37-1* and *vps28-2* backgrounds were obtained by crosses. FLS2-GFP was stably transformed into *vps28-2* mutants or crossed. RFP-VPS28-2 lines were generated in wild type and FLS2-GFP plants by stable transformation as described previously [50]. Constructs used in this study were obtained by PCR amplifying *VPS37-1* (AT3G53120), *VPS28-2* (AT4G05000) and *ELC* (AT3G12400) from Col-0 cDNA (Table S1) and cloned into pGWB binary vectors [51] using Gateway (Invitrogen). All constructs were confirmed by sequencing. *Nicotiana benthamiana* plants were grown under 16 hours of light at 24°C and 45–65% humidity.

### Transient transformation, staining and chemical treatments

FLS2-GFP transient transformation in *N. benthamiana*, and transient expression of RFP-ARA7/RabF2b by particle bombarding Arabidopsis leaves was done as described [3], [4]. FM4-64 staining in leaves was done as described previously [3]. Roots were incubated with FM4-64 for 30 min at 4°C to ensure simultaneous staining of the plasma membrane followed by 2 h at RT before imaging. Wortmannin and BFA treatments were done as reported before [3]. For combined treatments, FLS2-GFP × ARA6/RabF1-RFP plants were treated with 10 µM flg22 for 45 min to allow the internalization of the activated receptor into late endosomes, followed by 30 µM Wortmannin treatment for 2 h before imaging.

### Confocal microscopy and image analysis

Standard confocal microscopy was performed using the Leica SP5 microscope (Leica, Germany) and high-throughput confocal imaging was performed using the Opera microscope (PerkinElmer, Germany) as described previously [52]. Fluorescence intensity measurements were done by using the Leica SP5 software. A line was defined as region of interest (ROI) that transsected endosomal compartments and the fluorescence intensity of was determined per pixel along the ROI.

### Pathogen inoculation and growth assays

Bacterial inoculation assays were performed as previously described [53]. Briefly, *Pto* DC3000 was sprayed onto leaf surface at 10^8^ cfu/ml. Disease symptoms and bacterial numbers were scored at 1 and 4 days post inoculation. Surface-sterilized leaf disks from two leaves and at least four plants per genotype were excised and subjected for extraction to determine bacterial titers.

### Bioassays for PAMP-induced responses

ROS assays were performed as described previously [31]. Briefly, 16 leaf discs were excised per genotype of four-week-old plants and triggered with 1 µm flg22. ROS was measured with a Varioskan multiplate reader (Thermo Fisher Scientific, USA) for 35 min. MAPK activation was detected by immunoblot analysis using anti-phospho p44/p42 MAPK as previously described [54]. Inhibition of seedling growth was measured as previously described [54]. For callose induction, flg22 were applied at 1 µM for 24 hrs, and callose deposits were stained with aniline blue and visualized as described before [55]. Images were taken with the Axiophot microscope (Zeiss, Germany) and quantification of callose deposits was done using the Acapella software [56]. Flg22-triggered stomatal closure was essentially done as described previously [31], images were taken with the Opera microscope (PerkinElmar, Germany), and stomatal apertures were measured as the width/length ratio using the Acapella software.

### Immunoblot analysis and co-immunoprecipitation

Immunoblot analysis with the indicated antibodies was performed as described before [53]. Pull-down experiments were carried out as previously reported [57] with the following modifications: Transient transformed *N. benthamiana* leaves were infiltrated with flg22 solution (43), and subjected to protein extraction in 50 mM Tris-HCl, pH 7.5; 150 mM NaCl; 10% glycerol, 2 mM EDTA, 5 mM DTT, 1% Triton ×100; 1% (vol/vol) protease inhibitor mixture (Sigma). Following filtration through Miracloth (Calbiochem) and centrifugation at 8000 rpm and 16.000 rpm each for 15 min, 5 µl per g fresh weight of GFP-Trap coupled to agarose beads (Chromotek) were added to the supernatant and incubated for 2 hours, washed four times, boiled for 5 min at 65°C in extraction buffer and subjected to immunoblot analysis.

### Accession numbers

*vps37-1*.1 (SAIL_97_H04), *vps37-1*.2 (SALK_042859), *VPS37-1* (At3g53120), *vps28-2* (SALK_040274), *VPS28-2* (AT4G05000).

## Supporting Information

Figure S1 Homotypic fusions of MVBs by RFP-ARA7/RabF2b ectopic expression and prolonged Wortammin treatment. (*A, B*) Standard confocal micrographs show wild type epidermal cells transiently expressing RFP-ARA7/RabF2b. Cells with different levels of ectopic RFP-ARA7/RabF2b are represented; bar = 10 µm. (*C, D)* Detail images of RFP-ARA7/RabF2b as indicated by white boxes; bar = 1 µm. (*C*) Detail image of normal size MVB. (*D*) Detail image of the ring-like structure of enlarged MVBs. (*E, F*) Confocal micrographs show ARA6/RabF1-RFP transgenic leaf epidermal cells untreated (*E*) and treated with 30 µm Wortmannin for 3 h (*F*); bar = 10 µm. (*G, H*) Detail images of ARA6/RabF1-RFP as indicated by white boxes; bar = 1 µm. (*G*) Detail image of normal size MVB. (*H*) Detail image of the ring-like structure of enlarged MVBs. Transections across endosomes used for fluorescence intensity measurements are indicated by white lines. The histograms show RFP-ARA7/RabF1 and ARA6/RabF1-RFP fluorescent intensities depicted by red lines, respectively. Representative images of two experiments are shown.
(DOC)

Figure S2 VPS28-2 co-localizes with FLS2 at endosomal compartments and is required for immunity against *Pto* DC3000 infection but not for flg22-triggered ROS generation. (*A*) Standard confocal micrographs show Arabidopsis cotyledon epidermal cells of FLS2-GFP × RFP-VPS28-2 transgenic lines treated with 10 µM flg22 for 40 min. FLS2-GFP co-localizing with RFP-VPS28-1 endosomes are indicated by arrows. Inset pictures show FLS2-GFP endosomes co-localizing with RFP-VPS28-2 compartments (indicated by white boxes); bar = 10 µm. (*B*) Four weeks-old plants of the indicated genotypes were surface inoculated with *Pto* DC3000 and bacterial multiplication was monitored at 4 dpi. Shown are mean values +/− SE; n = 8; letters indicate significant differences p<0.01 based on ANOVA and Tukey's honestly test. (*C*) Stomatal apertures were measured following treatments with water (mock), 10 µM flg22, 5 µM ABA for 90 min. Bars represent mean values +/− SE; n>138<268 stomata. Statistical analysis is based on ANOVA and Tukey's honestly test and letters indicate statistical significance of p<0.05. (*D*) ROS generation in leaf discs of four weeks-old plants of the indicated genotypes triggered by 10 µM flg22 over time. Error bars represent mean values +/− SE; n = 16. (*E*) Quantification of FLS2-GFP endosomal numbers per image area upon treatment with 10 µM flg22 at the indicated times and genotypes. Independent transformants in T4 generation are indicated by numbers; homozygous crossed plants were used in F4 generation. Error bars represent mean values +/− SE; n>20>144 images. (*F*) Immunoblot detection of endogenous FLS2 and transgenic FLS2-GFP protein accumulation in homozygous Col-0, transformed and crossed *vps28-2* plants. Coomassie brilliant blue (CBB) is used as loading control. These experiments have been repeated three times with the same conclusion.
(DOC)

Figure S3 Effects of *vps37-1* mutants on flg22-triggered responses and endosomal trafficking. (*A*) FLS2 accumulation and MAPK activation upon application of 10 µM flg22 for the indicated times. Bands representing FLS2 and active forms of MPK3, 4, and 6 are indicated. Coomassie brilliant blue (CBB) is used as loading control. (*B*) Quantification of aniline blue stained callose deposits per cotyledon of the indicated genotypes treated with water (mock) or 10 µM flg22 for 10 hrs. Error bars represent mean values +/− SE; n>10<20. (*C*) Flg22-induced FLS2-GFP co-localizes with RFP-ARA7/RabF2b at endosomes in *vps37-1* mutants. High throughput confocal micrographs show maximal projections of 20 optical sections of leaf epidermal cells of Arabidopsis crossed lines stably expressing FLS2-GFP and RFP-ARA7/RabF2b in *vps37-1* mutant background treated with 10 µM flg22 for 80 min. Overlay image shows co-localization between FLS2-GFP and RFP-ARA7/RabF2b at vesicles; bar = 10 µm. Detail image of FLS2-GFP and RFP-ARA7/RabF2b co-localization as indicated by white boxes. (*D*) Standard confocal micrographs show Arabidopsis cotyledon epidermal cells of FLS2-GFP transgenic *vps37-1* mutant lines stained with FM4-64 after treatment with BFA in the absence or presence of 10 µM flg22 for 40 min. FLS2-GFP aggregation at FM4-64 stained BFA-bodies is indicated with arrows. Flg22-induced FLS2-GFP endosomes are indicated with arrowheads. Inset picture shows FLS2-GFP endosomes localizing around the BFA-body; bar = 10 µm. (*E*) Standard confocal micrographs show Arabidopsis root epidermal cells of wild type and *vps37-1* mutant plants stained with FM4-64 for 2 hrs. FM4-64 labelling of the tonoplast is visible around the nucleus, indicated by arrows; bar = 10 µm.
(DOC)

Figure S4 Molecular characterization of *vps28-2* and *vps37-1* T-DNA insertion mutants. (*A*) Semi-quantitative RT-PCR of *VPS37-1* expression in the indicated genotypes and position of the T-DNA insertions in *vps37-1.1* and *vps37-1.2* lines. (*B*) Semi-quantitative RT-PCR of *VPS28-2* expression in the indicated genotypes and position of the T-DNA insertions in the *vps28-21* line. Amplification of *ACTIN2* is shown as control.
(DOC)

Table S1 List of primers used in this study. qPCR, quantitative PCR; for, forward; rev, reverse, LP left border primer; RP right border primer; GW, Gateway.
(DOC)
