# Supplementary material for: ESCRT-I Mediates FLS2 Endosomal Sorting and Plant Immunity
Source: PLoS Genet. 2013 Dec 26;9(12):e1004035. doi: 10.1371/journal.pgen.1004035 (PMC3873229; doi:10.1371/journal.pgen.1004035)
Supplement: Figure S1 — Homotypic fusions of MVBs by RFP-ARA7/RabF2b ectopic expression and prolonged Wortammin treatment. (A, B) Standard confocal micrographs show wild type epidermal cells transiently expressing RFP-ARA7/RabF2b. Cells with different levels of ectopic RFP-ARA7/RabF2b are represented; bar = 10 µm. (C, D) Detail images of RFP-ARA7/RabF2b as indicated by white boxes; bar = 1 µm. (C) Detail image of normal size MVB. (D) Detail image of the ring-like structure of enlarged MVBs. (E, F) Confocal micrographs show ARA6/RabF1-RFP transgenic leaf epidermal cells untreated (E) and treated with 30 µm Wortmannin for 3 h (F); bar = 10 µm. (G, H) Detail images of ARA6/RabF1-RFP as indicated by white boxes; bar = 1 µm. (G) Detail image of normal size MVB. (H) Detail image of the ring-like structure of enlarged MVBs. Transections across endosomes used for fluorescence intensity measurements are indicated by white lines. The histograms show RFP-ARA7/RabF1 and ARA6/RabF1-RFP fluorescent intensities depicted by red lines, respectively. Representative images of two experiments are shown. (DOC) [file pgen.1004035.s001.doc]

**Figure S1**

**Figure S1. Homotypic fusions of MVBs by RFP-ARA7/RabF2b ectopic expression and prolonged Wortammin treatment.** (*A, B*) Standard confocal micrographs show wild type epidermal cells transiently expressing RFP-ARA7/RabF2b. Cells with different levels of ectopic RFP-ARA7/RabF2b are represented; bar = 10 µm. (*C, D)* Detail images of RFP-ARA7/RabF2b as indicated by white boxes; bar = 1 µm. (*C*) Detail image of normal size MVB. (*D*) Detail image of the ring-like structure of enlarged MVBs. (*E, F*) Confocal micrographs show ARA6/RabF1-RFP transgenic leaf epidermal cells untreated (*E*) and treated with 30 μm Wortmannin for 3 h (*F*); bar = 10 µm. (*G, H*) Detail images of ARA6/RabF1-RFP as indicated by white boxes; bar = 1 µm. (*G*) Detail image of normal size MVB. (*H*) Detail image of the ring-like structure of enlarged MVBs. Transections across endosomes used for fluorescence intensity measurements are indicated by white lines. The histograms show RFP-ARA7/RabF1 and ARA6/RabF1-RFP fluorescent intensities depicted by red lines, respectively. Representative images of two experiments are shown.
