# Supplementary material for: ESCRT-I Mediates FLS2 Endosomal Sorting and Plant Immunity
Source: PLoS Genet. 2013 Dec 26;9(12):e1004035. doi: 10.1371/journal.pgen.1004035 (PMC3873229; doi:10.1371/journal.pgen.1004035)
Supplement: Figure S2 — VPS28-2 co-localizes with FLS2 at endosomal compartments and is required for immunity against Pto DC3000 infection but not for flg22-triggered ROS generation. (A) Standard confocal micrographs show Arabidopsis cotyledon epidermal cells of FLS2-GFP × RFP-VPS28-2 transgenic lines treated with 10 µM flg22 for 40 min. FLS2-GFP co-localizing with RFP-VPS28-1 endosomes are indicated by arrows. Inset pictures show FLS2-GFP endosomes co-localizing with RFP-VPS28-2 compartments (indicated by white boxes); bar = 10 µm. (B) Four weeks-old plants of the indicated genotypes were surface inoculated with Pto DC3000 and bacterial multiplication was monitored at 4 dpi. Shown are mean values +/− SE; n = 8; letters indicate significant differences p<0.01 based on ANOVA and Tukey's honestly test. (C) Stomatal apertures were measured following treatments with water (mock), 10 µM flg22, 5 µM ABA for 90 min. Bars represent mean values +/− SE; n>138<268 stomata. Statistical analysis is based on ANOVA and Tukey's honestly test and letters indicate statistical significance of p<0.05. (D) ROS generation in leaf discs of four weeks-old plants of the indicated genotypes triggered by 10 µM flg22 over time. Error bars represent mean values +/− SE; n = 16. (E) Quantification of FLS2-GFP endosomal numbers per image area upon treatment with 10 µM flg22 at the indicated times and genotypes. Independent transformants in T4 generation are indicated by numbers; homozygous crossed plants were used in F4 generation. Error bars represent mean values +/− SE; n>20>144 images. (F) Immunoblot detection of endogenous FLS2 and transgenic FLS2-GFP protein accumulation in homozygous Col-0, transformed and crossed vps28-2 plants. Coomassie brilliant blue (CBB) is used as loading control. These experiments have been repeated three times with the same conclusion. (DOC) [file pgen.1004035.s002.doc]

**Figure S2. VPS28-2 co-localizes with FLS2 at endosomal compartments and is required for immunity against *Pto* DC3000 infection but not for flg22-triggered ROS generation.** (*A*) Standard confocal micrographs show Arabidopsis cotyledon epidermal cells of FLS2-GFP x RFP-VPS28-2 transgenic lines treated with 10 µM flg22 for 40 min. FLS2-GFP co-localizing with RFP-VPS28-1 endosomes are indicated by arrows. Inset pictures show FLS2-GFP endosomes co-localizing with RFP-VPS28-2 compartments (indicated by white boxes); bar = 10 µm. (*B*) Four weeks-old plants of the indicated genotypes were surface inoculated with *Pto* DC3000 and bacterial multiplication was monitored at 4 dpi. Shown are mean values +/- SE; n = 8; letters indicate significant differences p < 0.01 based on ANOVA and Tukey’s honestly test. (*C*) Stomatal apertures were measured following treatments with water (mock), 10 µM flg22, 5 µM ABA for 90 min. Bars represent mean values +/- SE; n > 138 < 268 stomata. Statistical analysis is based on ANOVA and Tukey’s honestly test and letters indicate statistical significance of p < 0.05. (*D*) ROS generation in leaf discs of four weeks-old plants of the indicated genotypes triggered by 10 µM flg22 over time. Error bars represent mean values +/- SE; n = 16. (*E*) Quantification of FLS2-GFP endosomal numbers per image area upon treatment with 10 µM flg22 at the indicated times and genotypes. Independent transformants in T4 generation are indicated by numbers; homozygous crossed plants were used in F4 generation. Error bars represent mean values +/- SE; n > 20 > 144 images. (*F*) Immunoblot detection of endogenous FLS2 and transgenic FLS2-GFP protein accumulation in homozygous Col-0, transformed and crossed *vps28-2* plants. Coomassie brilliant blue (CBB) is used as loading control. These experiments have been repeated three times with the same conclusion.
