# Supplementary material for: ESCRT-I Mediates FLS2 Endosomal Sorting and Plant Immunity
Source: PLoS Genet. 2013 Dec 26;9(12):e1004035. doi: 10.1371/journal.pgen.1004035 (PMC3873229; doi:10.1371/journal.pgen.1004035)
Supplement: Figure S3 — Effects of vps37-1 mutants on flg22-triggered responses and endosomal trafficking. (A) FLS2 accumulation and MAPK activation upon application of 10 µM flg22 for the indicated times. Bands representing FLS2 and active forms of MPK3, 4, and 6 are indicated. Coomassie brilliant blue (CBB) is used as loading control. (B) Quantification of aniline blue stained callose deposits per cotyledon of the indicated genotypes treated with water (mock) or 10 µM flg22 for 10 hrs. Error bars represent mean values +/− SE; n>10<20. (C) Flg22-induced FLS2-GFP co-localizes with RFP-ARA7/RabF2b at endosomes in vps37-1 mutants. High throughput confocal micrographs show maximal projections of 20 optical sections of leaf epidermal cells of Arabidopsis crossed lines stably expressing FLS2-GFP and RFP-ARA7/RabF2b in vps37-1 mutant background treated with 10 µM flg22 for 80 min. Overlay image shows co-localization between FLS2-GFP and RFP-ARA7/RabF2b at vesicles; bar = 10 µm. Detail image of FLS2-GFP and RFP-ARA7/RabF2b co-localization as indicated by white boxes. (D) Standard confocal micrographs show Arabidopsis cotyledon epidermal cells of FLS2-GFP transgenic vps37-1 mutant lines stained with FM4-64 after treatment with BFA in the absence or presence of 10 µM flg22 for 40 min. FLS2-GFP aggregation at FM4-64 stained BFA-bodies is indicated with arrows. Flg22-induced FLS2-GFP endosomes are indicated with arrowheads. Inset picture shows FLS2-GFP endosomes localizing around the BFA-body; bar = 10 µm. (E) Standard confocal micrographs show Arabidopsis root epidermal cells of wild type and vps37-1 mutant plants stained with FM4-64 for 2 hrs. FM4-64 labelling of the tonoplast is visible around the nucleus, indicated by arrows; bar = 10 µm. (DOC) [file pgen.1004035.s003.doc]

**Figure S3. Effects of *vps37-1* mutants on flg22-triggered responses and endosomal trafficking.** (*A*) FLS2 accumulation and MAPK activation upon application of 10 µM flg22 for the indicated times. Bands representing FLS2 and active forms of MPK3, 4, and 6 are indicated. Coomassie brilliant blue (CBB) is used as loading control. (*B*) Quantification of aniline blue stained callose deposits per cotyledon of the indicated genotypes treated with water (mock) or 10 µM flg22 for 10 hrs. Error bars represent mean values +/- SE; n > 10 < 20. (*C*) Flg22-induced FLS2-GFP co-localizes with RFP-ARA7/RabF2b at endosomes in *vps37-1* mutants. High throughput confocal micrographs show maximal projections of 20 optical sections of leaf epidermal cells of Arabidopsis crossed lines stably expressing FLS2-GFP and RFP-ARA7/RabF2b in *vps37-1* mutant background treated with 10 µM flg22 for 80 min. Overlay image shows co-localization between FLS2-GFP and RFP-ARA7/RabF2b at vesicles; bar = 10 µm. Detail image of FLS2-GFP and RFP-ARA7/RabF2b co-localization as indicated by white boxes. (*D*) Standard confocal micrographs show Arabidopsis cotyledon epidermal cells of FLS2-GFP transgenic *vps37-1* mutant lines stained with FM4-64 after treatment with BFA in the absence or presence of 10 µM flg22 for 40 min. FLS2-GFP aggregation at FM4-64 stained BFA-bodies is indicated with arrows. Flg22-induced FLS2-GFP endosomes are indicated with arrowheads. Inset picture shows FLS2-GFP endosomes localizing around the BFA-body; bar = 10 µm. (*E*) Standard confocal micrographs show Arabidopsis root epidermal cells of wild type and *vps37-1* mutant plants stained with FM4-64 for 2 hrs. FM4-64 labelling of the tonoplast is visible around the nucleus, indicated by arrows; bar = 10 µm.
