# Supplementary material for: ESCRT-I Mediates FLS2 Endosomal Sorting and Plant Immunity
Source: PLoS Genet. 2013 Dec 26;9(12):e1004035. doi: 10.1371/journal.pgen.1004035 (PMC3873229; doi:10.1371/journal.pgen.1004035)
Supplement: Figure S4 — Molecular characterization of vps28-2 and vps37-1 T-DNA insertion mutants. (A) Semi-quantitative RT-PCR of VPS37-1 expression in the indicated genotypes and position of the T-DNA insertions in vps37-1.1 and vps37-1.2 lines. (B) Semi-quantitative RT-PCR of VPS28-2 expression in the indicated genotypes and position of the T-DNA insertions in the vps28-21 line. Amplification of ACTIN2 is shown as control. (DOC) [file pgen.1004035.s004.doc]

**Figure S4. Molecular characterization of *vps28-2* and *vps37-1* T-DNA insertion mutants.** (*A*) Semi-quantitative RT-PCR of *VPS37-1* expression in the indicated genotypes and position of the T-DNA insertions in *vps37-1.1* and *vps37-1.2* lines. (*B*) Semi-quantitative RT-PCR of *VPS28-2* expression in the indicated genotypes and position of the T-DNA insertions in the *vps28-21* line. Amplification of *ACTIN2* is shown as control.
