# Supplementary material for: ESCRT-I Mediates FLS2 Endosomal Sorting and Plant Immunity
Source: PLoS Genet. 2013 Dec 26;9(12):e1004035. doi: 10.1371/journal.pgen.1004035 (PMC3873229; doi:10.1371/journal.pgen.1004035)
Supplement: Table S1 — List of primers used in this study. qPCR, quantitative PCR; for, forward; rev, reverse, LP left border primer; RP right border primer; GW, Gateway. (DOC) [file pgen.1004035.s005.doc]

**Table S1. List of used primers in this study.**

| ACT2_qPCR_for | GGTAACATTGTGCTCAGTGGTGG |
| --- | --- |
| ACT2_qPCR_rev | AACGACCTTAATCTTCATGCTGC |
| TUB_qPCR_for | AGGGAAACGAAGACAGCAAG |
| TUB_qPCR_rev | GCTCGCTAATCCTACCTTTGG |
| VPS28-2_GW_for | GGGGACAAGTTTGTACAAAAAAGCAGGCTTAATGATGGAGGTCAAATTATGGAACGAC |
| VPS28-2_GW_rev | GGGGACCACTTTGTACAAGAAAGCTGGGTATTAATTACCAGCTTTAGGCAAAGCTGCC |
| VPS28-2_LP_for | TCAAATTAATAAAATTTCACGGTCC |
| VPS28-2_RP_rev | GACAAACGCGAAAGAGAGATG |
| VPS37-1.1_LP_for | TGGAGGATCTGATGGAGAATG |
| VPS37-1.1_RP_rev | TCCTGAGTTCATCCACGCTAC |
| VPS37-1.2_LP_for | AAGAAGCTTCCTGAGGACGAG |
| VPS37-1.2_RP_rev | TTCGCGATTGGTATACCTGAC |
| VPS37-1_GW_for | GGGGACAAGTTTGTACAAAAAAGCAGGCTTAATGTTCAATTTCTGGGGATC |
| VPS37-1_GW_rev | GGGGACCACTTTGTACAAGAAAGCTGGGTAAATGTTTGACGTTTTAGC |
| VPS37-1_qPCR_for | GGATCAAAAGACCAACAACAAGGGC |

qPCR, quantitative PCR; for, forward; rev, reverse, LP left border primer; RP right border primer; GW, Gateway
